# Small Changes in Gene Expression of Targeted Osmoregulatory Genes When Exposing Marine and Freshwater Threespine Stickleback (*Gasterosteus aculeatus*) to Abrupt Salinity Transfers

**DOI:** 10.1371/journal.pone.0106894

**Published:** 2014-09-29

**Authors:** Annette Taugbøl, Tina Arntsen, Kjartan Østbye, Leif Asbjørn Vøllestad

**Affiliations:** 1 Centre for Ecological and Evolutionary Synthesis (CEES), Department of Biosciences, University of Oslo, Blindern, Norway; 2 Hedmark University College, Department of Forestry and Wildlife Management, Campus Evenstad, Elverum, Norway; Ecole Normale Supérieure de Lyon, France

## Abstract

Salinity is one of the key factors that affects metabolism, survival and distribution of fish species, as all fish osmoregulate and euryhaline fish maintain osmotic differences between their extracellular fluid and either freshwater or seawater. The threespine stickleback (*Gasterosteus aculeatus*) is a euryhaline species with populations in both marine and freshwater environments, where the physiological and genomic basis for salinity tolerance adaptation is not fully understood. Therefore, our main objective in this study was to investigate gene expression of three targeted osmoregulatory genes (Na^+^/K^+^-ATPase (ATPA13), cystic fibrosis transmembrane regulator (CFTR) and a voltage gated potassium channel gene (KCNH4) and one stress related heat shock protein gene (HSP70)) in gill tissue from marine and freshwater populations when exposed to non-native salinity for periods ranging from five minutes to three weeks. Overall, the targeted genes showed highly plastic expression profiles, in addition the expression of ATP1A3 was slightly higher in saltwater adapted fish and KCNH4 and HSP70 had slightly higher expression in freshwater. As no pronounced changes were observed in the expression profiles of the targeted genes, this indicates that the osmoregulatory apparatuses of both the marine and landlocked freshwater stickleback population have not been environmentally canalized, but are able to respond plastically to abrupt salinity challenges.

## Introduction

The ability to respond rapidly to environmental change is beneficial in variable environments, making phenotypically plastic organisms better adapted in unstable and unpredictable environments [1]. The capacity of an organism to respond to its environment is facilitated by the environmentally induced alteration of gene and protein expression. While the evolution of plasticity depends on the trait(s) in question and the source of environmental variation, there is a general acceptance that the ability to be plastic may be constrained by a variety of costs underlying the plastic responses [2]. As such, evolutionary theory predicts loss of plasticity after periods of environmental stability, when environmental constancy eliminates or weakens the source of selection that was formerly important for its maintenance, given that the cost for the trait is high [3], or through environmentally induced genetic assimilation [4] which reduces the environmental influence on trait expression.

Phenotypic plasticity of a trait is generally assumed to be under selection when a single organism is exposed to several environments during its lifetime which each select for different trait values. Most fish species are stenohaline, living either in fresh or salt water [5], [6] where they are exposed to the same type of osmoregulatory challenge during their lifetime. For fish living in marine waters, the concentration of ions is much higher in the water compared to the environment inside the cell, and surrounding ions diffuse into the cell while water is lost. The situation is reversed for a freshwater fish, where the surroundings are ion depleted, making the fish passively loose ions and gain water. In order to maintain a relatively stable internal osmotic environment, fish counteract these effects by a variety of specialized physiological mechanisms, mainly in the gills [7] and the kidney [8], and these genetic adaptations can limit movement between salinities. Only a very limited number of species are truly euryhaline [6], being able to osmoregulate in a wide variety of salinity environments. Even fewer can tolerate extreme changes in osmolality over short time scales, such as the killifishes (*Fundulus spp.*) [9] and the threespine stickleback (*Gasterosteus aculeatus*) [10], [11].

The threespine stickleback (order, Gasterosteiformes, family Gasterosteidae; hereafter stickleback) is a small fish that was originally a marine species [12]. However, since the last glaciation, sticklebacks have colonized a large number of brackish and freshwater systems throughout the northern hemisphere and are now occupying an extremely wide haloniche [11], [13]. Many of the newly formed freshwater populations have become landlocked due to the isostatic uplifting of the land following deglaciation, and the stickleback in these habitats have consequently been separated from the sea for thousands of years. If the costs of having a plastic osmoregulatory machinery is high, it is expected that these landlocked freshwater stickleback populations should have lost the ability to osmoregulate efficiently in saltwater. However, studies suggest that freshwater populations of stickleback still possess the osmoregulatory machinery enabling them to handle abrupt changes in salinity [10], [14], despite having been separated from the marine environment for up to 10–18 000 years [12]. This indicates that during adaptation to freshwater environments, the osmoregulatory physiology of landlocked sticklebacks has not been environmentally assimilated, or alternatively, the functionality of the osmoregulatory apparatus and its genomic architecture may not be open for selective change due to pleiotropic gene-interactions and is thus expected to remain similar in freshwater and marine populations.

**Figure 1:**
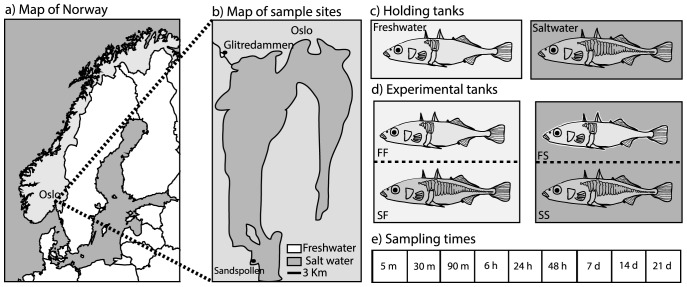
Study area and experimental design. a) Map of Norway showing the position of the sampling sites b) Locations of the two sampling sites, Glitredammen (freshwater) and Sandspollen (marine) c) Wild caught fish from both sampling locations were taken into the lab and placed in holding tanks of their native salinity for a minimum of three weeks. After acclimation, two groups of eight fish from both populations were exposed to either saltwater (30 PSU) or freshwater (0 PSU). The exposure tanks were divided in two by a perforated wall, so that both populations could be exposed to the same water quality at the same time d) Exposure times before the fish was collected and gill tissue was sampled.

One way to test if fish are adapted to a particular haloniche is to expose individuals to salinity challenges by transferring individuals from the original salinity to a test-salinity, tracking the expression of relevant genes through time. Earlier experiments show that stickleback easily tolerate transfers from freshwater to fully marine salinity, as well as the reverse [10], [14]. However, it is not clear if the same osmoregulatory machinery is functioning at all times. The aim of this study was to assess the effect of experimental manipulation of salinity on the expression of genes important for osmoregulation. For this purpose, we collected adult fish from one marine and one freshwater site and exposed fish from each population to either 0 or 30 PSU (practical salinity units) for periods varying from 5 minutes to 3 weeks. The expression of four candidate genes was then followed through time (Fig. 1); three of the included genes are related to ion-pumps recognized to be under selection in marine-freshwater gradients (Na^+^/K^+^-ATPase (ATP1A3), cystic fibrosis transmembrane regulator (CFTR), voltage gated potassium channel gene (KCNH4)), and one is a stress related heat shock protein gene gene (HSP70), also associated with osmotic stress.

The objective of the study was to i) assess how target genes were expressed in the native salinity (assuming adaptation), ii) assess how target genes were affected when freshwater adapted fish were exposed to saltwater and iii) assess how target genes were affected when saltwater adapted fish were exposed to freshwater. As the osmoregulatory challenges are opposite in freshwater and marine environments, with ion secretion needed in saltwater and ion uptake needed in freshwater, we expect that osmoregulatory genes upregulated in freshwater will be downregulated in saltwater, and vice versa. We further expect the stress-related gene to have elevated expression levels in the beginning of the exposure for both groups due to handling and the physiological challenges associated with changing gene expression.

## Materials and Methods

### Fish and maintenance conditions

Adult stickleback were captured at two locations near Oslo, Norway (Figure 1a, b), during May and June 2010 and 2011. Fish from the marine population are known to breed there, and are not migratory, as many populations are known to be elsewhere [15], [16], [17]. The marine site (Sandspollen; 59^o^ 39′ 58″N; 10^o^ 35′ 11″ E) has a salinity that fluctuates between 22–29 PSU, while the freshwater pond (Glitredammen; 59^o^ 55′ 53″N; 10^o^ 29′ 55″ E; elevation 82.8 m above sea level) is stable at 0 PSU. The marine fish is comprised only of the completely plated morph (having a full row of lateral plates along its body flank), while the freshwater population only has the low plated morph (with lateral plates in the front region only). The two locations are geographically isolated by approximately 35 km by shortest distance through water, where about 8.5 km is through the river Sandvikselva that contains several steep waterfalls and dams. The age of the lake has been estimated at 7800 years before present using the program Sealevel32 [18]. The program uses information on postglacial land uplift and water level rise to estimate lake age. Downstream movement of fish from Glitredammen is possible, but upstream movement from the sea is impossible.

After capture, the fish were transported to the aquarium facility at the University of Oslo and acclimated to holding conditions in their native salinity for minimum three weeks prior to the experiment. Two glass holding-tanks (500L) with either salt (30 PSU) or fresh water (0 PSU) were used for acclimation (Figure 1c), using biologically activated canister filters (EHEIM professional 3600), and UV-filtration. The acclimation tanks were covered with black plastic in front and on the sides to reduce visual stress. Further, to reduce potential male nesting behavior, the tanks were not equipped with any environmental enrichment, leaving the tanks free of sand and vegetation. The temperature in the tanks was maintained at room temperature (approx. 20°C) and the light regime was set at a 12∶12 light:dark cycle. The fish were fed two times a day with frozen red bloodworms throughout the acclimation and exposure period.

### Experimental design and protocol

The experimental setup consisted of 80 L tanks; the tanks were either filled with 0 PSU water or with 30 PSU water (Figure 1c), and covered with black sheets to reduce visual stress. A grey plastic wall divided each experimental tank into two 40 L compartments, where perforation ensured water movement between compartments (Figure 1d).

At the start of an experiment, 8 fish that appeared healthy were collected from each holding tank and placed directly in either the 30 or the 0 PSU experimental tank. Saltwater fish were therefore tested in either saltwater (SS; control) or in freshwater (SF). The freshwater fish were also tested in saltwater (FS) or in freshwater (FF; control) (Figure 1d).

The fish were exposed for different time periods, lasting between 5 minutes and 3 weeks (Figure 1e). The time periods were selected to cover short-term effects as well as long-term changes in gene expression. After each experiment, the fish were quickly netted out of the experimental tanks, immediately killed by a swift blow to the head and was directly processed for tissue collection.

### Ethics statement

The experiment was approved by the Norwegian Animal experimentation and care committee (permit no ID 2705) and all efforts were made to minimize suffering.

### Candidate gene expression

Candidate genes for osmoregulation were selected based on published studies on divergence in gene expression between marine and freshwater sticklebacks [19], studies identifying outlier regions in DNA sequences between marine and freshwater sticklebacks [20], and preliminary Illumina RNA-sequencing results (Table 1).

**Table 1:** Primers used for qPCR expression analysis of threespine stickleback genes.

Target	Gene name	Ensembl gene ID	Primer sequences	°C	E (%)	Reference
ATP1A3b	ATPase, Na+/K+ transporting, alpha 3b polypeptide	ENSGACG00000009524	F: AGCCGAGATCCCCTTCAACTCCA	60	99.07	*This study*
			R: GCTCCTTCCCCTGCACCAGGA			
CFTR	Cystic fibrosis transmembrane conductance regulator	ENSGACG00000009039	F: GCAGGCCTCTTCTTCACCAA	58	98.51	McCairns et al. (2009)
			R: TCCAGATAGAGGCTGATGTTCTTG			
KCNH4	Potassium voltage-gated channel subfamily H member 4	ENSGACG00000008648	F: CACAGTGACCTCTCTGGTGC	60	99.29	*This study*
			R: AGACATGAGCAGGGTCAGGA			
HSP70	Heat shock protein 70	ENSGACG00000013048	F: ATCGGTATTGACCTGGGCAC	60	99.20	*This study*
			R: GGTATCGGTGAACGCCACAT			
**Reference**						
EF1α	Elongation factor 1	ENSGACG00000002182	F: CATTGTCACTTACCTGAATCACATGA	60	99.26	McCairns et al. (2009)
			R: TGTGGCATTTAACAACATTTCCA			
GADPH	Glyceraldehyde-3-phosphate dehydrogenase	ENSGACG00000005864	F: CAAACCGTTGGTGACAGTATTTG	60	99.9	Sanago et al. (2011)
			R: GCACTGAGCATAAGGACACATCTAA			

The targeted Na^+^/K^+^-ATPase gene, ATP1A3, has displayed salinity dependent regulation in fish when acclimated to different salinities, including killifish, *Fundulus heteroclitus* [21]. ATP1A3 is a plasma membrane protein that helps the establishment and maintenance of the electrochemical gradients of sodium and potassium ions across the plasma membrane by coupling the exchange of two extracellular K+ ions for three intracellular Na+ ions to the hydrolysis of one molecule of ATP [22], thereby ensuring a relatively constant osmolarity of cells and blood plasma. The protein is powering salt secretion in saltwater fish and salt absorption in freshwater fish [23].

The cystic fibrosis transmembrane regulator, CFTR, is an apical membrane anion channel involved in chloride secretion, and establishes an electrical driving force for trans-epithelial sodium secretion that generate the osmotic driving force for water flow, yielding an isotonic secretory product. As a candidate gene for saltwater adaptation [24], previous studies have also shown an upregulation of chloride cells and CFTR expression in a Hawaiian goby (*Stenogobius hawaiiensis*) [25] and in killifish [26] exposed to salt water.

While able to tolerate a wide range of salinity, whole genome sequencing of marine and freshwater sticklebacks have identified several chromosomal regions that have undergone parallel selection after freshwater invasion, indicating adaptive divergence and evolutionary change across the marine-freshwater boundary [20]. One identified region differing between marine and freshwater sticklebacks was an inversion with alternative functional exons of the voltage gated potassium channel gene, KCNH4, on either side, suggesting marine and freshwater specific isoforms [20]. However, although small, parallel changes in the sequences of genes may result from similar selection pressure across environments [27], [28], [29], it is the functional gene products and its regulation through expression that gives rise to the phenotype. Therefore, when candidate regions or loci linked to adaptive divergence have been identified, the regions should be tested in function, such as their role in gene regulation in a relevant ecological setting. The primer pairs in this study does not distinguish between marine and freshwater isoforms as the spanning ends of the inversion are identical down to a few base-pairs and the mutations are seemingly located within introns of the gene.

Both the physical handling when fish are transferred between tanks and the changes in water salinity are stressful, thus a stress-related heat-shock protein, HSP70, known to be affected by osmotic stress [30] was also included in the study.

To control for variation in expression levels not due to the experimental treatment, we used two reference genes, Elongation factor 1 alpha (EF1α) and Gluceraldehyde-3-phosphate dehydrogenase (GADPH). EF1α has been used successfully as reference gene in a similar study on sticklebacks [19] as well as in other gene expression studies on fish [26]. GADPH is a commonly used reference gene and has been stably expressed in a wide array of studies spanning predator cues [31] to exposure to offshore produced water [32].

Gene specific primers for target genes CFTR and reference genes GADPH and EF1α were previously designed and optimized (Table 1). Primers for additional target genes (ATP1A3, KCNH4 and HSP70) were designed based on genetic sequences from the Enseml genome browser and NCBI Primer-Blast (Table 1).

### Tissue collection, RNA isolation, cDNA synthesis and qPCR

The gill plays an important role in the maintenance of blood ion and acid–base balance in both freshwater- and seawater acclimated fish [7], [33], [34]. After each fish was sacrificed, gill samples were immediately collected using sterilized tweezers and stored in RNA*later* (Ambion RNA, Life Technologies) according to the manufacturers protocol. The sampled fish were stored individually in 70% EtOH. The mRNA was isolated from the gill samples from each fish separately, using the mRNA direct kit (Invitrogen) as described by the manufacturer. The mRNA concentration and purity was quantified using Bioanalyzer (Agilent 2100 Bioanalyzer) and the Agilent RNA 6000 Pico Kit (Agilent Technologies) according to the protocol, and all samples were diluted down to 0.125 µg/µL before cDNA synthesis. The cDNA was prepared using the Superscript VILO cDNA synthesis kit (Invitrogen by Life Technologies) as described by the manufacturer, and the concentration was checked spectrophotometrically using Nanodrop (NanoDrop Teqnologies INC).

The cDNA concentration was diluted down to 15 ng/µL (±1.5 ng/µL) prior to qPCR amplification after testing for optimization (standard curves, two-fold serial dilutions on pooled cDNA) and association curves for each primer pair (all primer pairs tested on dilution curves at 58 and 60°C). Primer efficiencies were calculated using the formula E = (10^−1/slope^)-1. All primer pairs had efficiencies between 95–100% and presented a single product, confirmed with a melting curve.

The qPCR reaction was performed on a Lightcycler 480 (Roche) using SYBR Green PCR Master Mix (Roche). Each 20 µL reaction contained 5.0 µL of the optimized concentrated cDNA, 1.0 µL of each primer, 10.0 µL of SYBR Green and 3.0 µL H2O. The thermocycle program included an enzyme activation step for 5 min, followed by 45 cycles of 95°C for 10 s, 58/60°C for 20 s and 72°C for 20 s. After the amplification phase, a dissociation curve was generated to confirm the presence of a single amplicon. The individual samples were run on duplicated plates, along with three negative reverse-transcriptase controls and an eightfold serial dilution to calibrate plate variation between runs. The obtained cycle threshold (C_q_) values for the individuals were adjusted for plate efficiency, and duplicated reactions that differed by more than 0.5 C_q_ values were checked manually and removed from the analysis.

### Statistical analysis

The target gene C_q_ values were normalized using the mean of the two control genes. Both the EF1α and GADPH were relatively stably expressed across the various time points but did differ somewhat between treatments. The C_q_ values for GADPH were slightly higher in the SS and SF treatments than in the other treatments (F_3, 237_ = 6.64, P<0.001), but did not differ between treatments for EF1α (F_3, 235_ = 1.43, P = 0.236). The relative expression levels were expressed as the individuals normalized C_q_-values of the target transcript, and expressed relative to the mean values of a control group, here set to the 5 min exposure, for each treatment group (SS, SF, FF, FS). This method gives the fold change in expression relative to the control [35].

Variation in fold change of the expression of the different target genes was tested using general linear models. Each treatment group was tested at 9 different time points, where time can be classified both as a continuous variable (in minutes) as well as an ordinal factor (1–9). Preliminary analyses indicated that using continuous time was the better modeling approach and was therefore used in the model, expressed on a log-scale. To account for non-linear effects we included a squared term for time. The general model structure was thus:
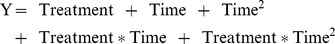
where treatment is the four different treatment types (SS, SF, FF, FS). The best (most parsimonious) model was selected using backward selection, using the Bayesian information criterion (BIC). BIC puts a heavier penalty on parameter number than the more commonly used Akaike information criterion (AIC).

## Results

In general all experimental fish handled the transfer to the experimental water qualities well, both when transferred to the control water quality (groups SS and FF) and to the treatment salinity (SF, FS). A total of 9 fish from the freshwater population died across treatments during the experiment (6 in FF and 3 in FS), whereas no marine fish died. A total of 288 sticklebacks were used throughout this study.

**Figure 2:**
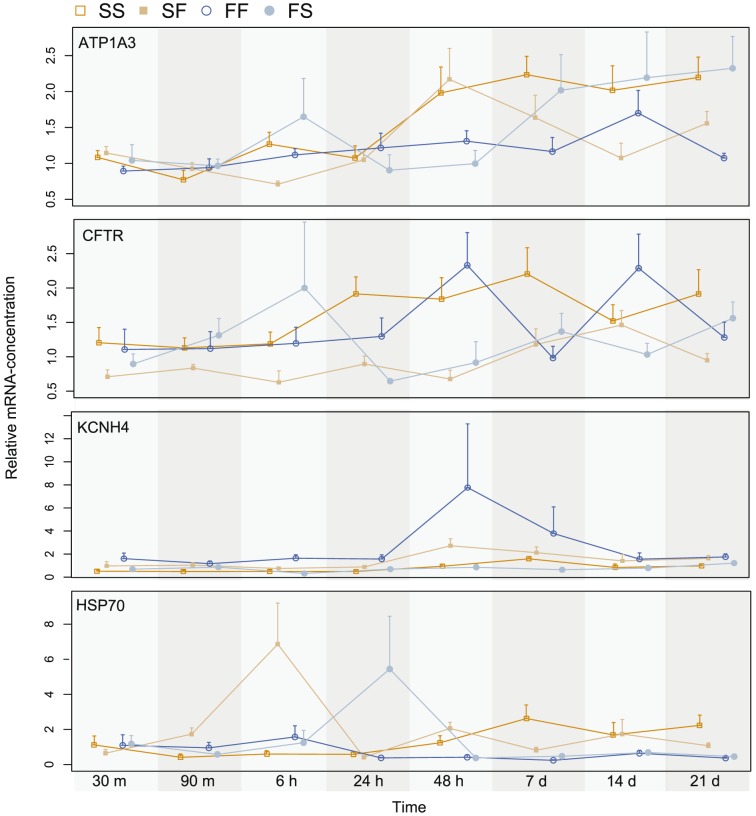
Relative mRNA-expression for the four targeted genes, ATP1A3, CFTR, KCNH4 and HSP70 in saltwater control (SS, dark orange), saltwater fish exposed to freshwater (SF, light orange), freshwater control (FF, dark blue) and freshwater fish exposed to saltwater (FS, light blue) for exposure periods relative to the 5 minute exposure. Values between 0–1 indicate lower expression, and values over 1 indicate higher expression relative to the expression at 5 minutes.

We used the gene expression levels at 5 minutes of exposure as the control against which all fold level changes in expression was compared. Overall there were only minor differences in C_q_ – levels among the various treatment groups for the target genes (Table 2, Figure 2, Appendix S1).

**Table 2:** Gene expression of four osmoregulatory genes (see Table 1) (C_q_-values; mean ± se) for marine and freshwater stickleback after 5 min exposure to either salt or freshwater.

Treatment	CFTR	ATP	KCNH4	HSP70
**SS**	1.15 3±0.009 (7)	1.111±0.009 (7)	1.415±0.013 (7)	1.272±0.020 (7)
**SF**	1.133±0.013 (4)	1.121±0.010 (6)	1.429±0.014 (6)	1.257±0.022 (6)
**FF**	1.158±0.009 (8)	1.126±0.008 (8)	1.413±0.012	1.196±0.019 (8)
**FS**	1.148±0.011 (6)	1.121±0.010 (6)	1.374±0.014 (6)	1.215±0.022 (6)
	F_3, 21_ = 0.836, P = 0.489	F_3, 23_ = 1.383, P = 0.273	F_3, 23_ = 2.836, P = 0.061	F_3, 23_ = 3.028, P = 0.050

Summary statistics from an analysis of variance for each gene is also given. Treatments are: SS (saltwater fish in saltwater), SF (saltwater fish in freshwater), FF (freshwater fish in freshwater) and FS (freshwater fish in saltwater).

The model that best explained variation in fold change in ATP-expression contained the ln-time and treatment factor (F_4, 204_ = 14.636; P<0.0001). Overall there was a tendency for the relative expression to increase with time, and the time-adjusted mean fold change was slightly larger for the SS and FS (SS: 1.547±0.093; FS: 1.555±0.105) than for the FF and SF treatments (SF: 1.283±0.094; FF: 1.193±0.105). To further examine how the fold-change varied with treatment we re-ran the analyses grouping the fish into those tested in freshwater (FF, SF) and those tested in saltwater (SS, FS). Overall, the best model using this model-structure fit better to the data than the best model using treatment group (BIC treatment group  = 478.0; BIC treatment water type  = 466.0). The best model contained the interaction with time and water treatment type.

The model that best explained variation in fold change in CFTR-expression contained the ln-time and treatment factor (F_4, 206_ = 13.041; P<0.0001). Overall there was a tendency for the relative expression to increase with time, and the time-adjusted mean fold change was larger for the two control treatments (SS: 1.592±0.087; FF: 1.427±0.099) than for the transfer treatments (SF: 0.903±0.088; FS: 1.155±0.094). The transfer treatments and the control treatments differed significantly (Tukey HSD post hoc test, P<0.05).

The model that best explained variation in fold change in KCNH-expression contained the ln-time (F_1,207_ = 14.937; P = 0.0001) and treatment factor (F_3,207_ = 14.593; P<0.0001). Overall there was a tendency for the relative expression to increase with time (0.087±0.023). To further examine how the fold-change varied with treatment we reran the analyses grouping the fish into those tested in freshwater (FF, SF) and those tested in saltwater (SS, FS). Overall the best model using this model structure fit better to the data than the best model using treatment group (BIC treatment group  = 527.1; BIC treatment water type  = 537.6). The best model contained time (F_1,209_ = 14.944; P<0.0001 and water treatment type (F_1,209_ = 43.995; P<0.0001). The KCNH-expression was significantly elevated in freshwater (1.488±0.079), whereas it was significantly decreased in saltwater (0.756±0.077).

The model that best explained variation in fold change in HSP70-expression only contained the treatment factor (F_3, 203_ = 2.688; P = 0.048). Overall, mean fold change was larger than one for fish originating from saltwater (SS: 1.286±0.181; SF: 1.526±0.182) whereas if was smaller than one for the fish originating from freshwater (FF: 0.865±0.209; FS: 0.902±0.205). The fish from freshwater and saltwater differed significantly in expression level (Tukey HSD post hoc test, P<0.05). However, despite the expression levels being quite different between the ecotypes, the explanatory power of the model was low (R^2^ = 0.038).

## Discussion

Teleost fishes maintain nearly constant internal osmotic concentration and have osmoregulatory machinery fine-tuned to the external salinity in either salt or fresh water. However, some species can tolerate a wide range of environmental salinity, also with only short or no acclimation. Our main objective in this study was to investigate the expression of three relevant osmoregulatory genes and one stress related gene in marine and freshwater threespine stickleback, when exposed to non-native salinity for various periods. The study showed that both populations were capable of handling a direct transfer to a new and very different salinity. Survival was high in all treatments and the variation in gene expression was relatively small. This suggests that the capacity for osmoregulation in a wide range of salinity regimes has not been lost in either population. And yet, interestingly, no pronounced changes were observed in the expression profiles of the genes targeted in this study. This suggests that the ability of these fish to reverse the direction of their osmoregulation has not been canalized or lost.

### Expression of ATP1A3 was elevated in saltwater

In this study, the ATP1A3 expression was lower in the FF treatment group at all time points, with a mean difference of 0.03 on an exponential scale compared to SS. Further, the SF group did show a weak down-regulation of ATP1A3 compared to SS and the expression levels stabilized around the FF values after approximately 24 hours. Comparing the FF and the FS, the FF had a consistently lower expression of ATP1A3, equally as the SS group.

As the Na^+^/K^+^-ATPase transporters both secrete and absorb salt in order to obtain a nearly constant internal osmotic concentration when in marine- and freshwater, respectively [7], it might be expected that the long-term expression of the protein stabilize on equal levels. Nevertheless, when a fish experiences a change in external osmolarity, the expression level is expected to shift in order to handle the novel osmotic and ionic stress: it is therefore surprising that we see so little change in the overall expression on the shorter time-scale in this study. Overall, the main finding of ATP1A3 being less expressed in freshwater is in accordance with most research on salmonid fish, where gill Na^+^/K^+^-ATPase activity is higher in seawater acclimated fish and decreases following migration into freshwater [36], [37]; other fish species also show this pattern, including sea bass, *Dicentrarchus labrax* [38] and flounder, *Platichthys flesus* [39]. However, yet other studies have shown that the expression levels for the equivalent ATP isoform in Atlantic salmon (*Salmo salar*) did not change as a result of freshwater exposure [40], or as in killifish, where ATP1A3 was up-regulated in freshwater [21]. Overall, previous reviews on fish osmoregulation state that the role of gill Na^+^/K^+^-ATPase is uncertain [41], unclear [7], or that the energy required for sodium uptake can be generated only by Na^+^/K^+^-ATPase [42]. It should however be noted that the cellular localization of the Na^+^/K^+^-ATPase transporter in this study is not known, and a histological analysis of the gene localization during a salinity challenge could provide additional information on the osmoregulatory function in stickleback.

### No major changes in CFTR expression between treatments

Overall, only small changes in CFTR expression were observed across the treatments in the present study. CFTR had a slightly higher expression level in SS compared to FF, but only in the early time-periods (5 min to 24 hours), where the overall expression was reduced in both treatments compared to T = 5 min. After the first day, the expression stabilizes for both groups. Transferring marine fish into freshwater (SF) lead to an overall reduced expression of CFTR compared to the SS treatment, but this trend was not consistent across all time points. The freshwater fish transferred to saltwater (FS) had a higher expression of CFTR compared to FF for the first 6 hours, but was generally stable across all time points, indicating no major change in expression.

CFTR is thought to be central in the ion excretion at the gills of marine fish, as it is involved in the passive transport of chloride ions [24], and as such the expression is expected to decrease following freshwater acclimation. Anticipated increased expression of CFTR in apical membranes in response to transfer to saltwater have been illustrated in Atlantic salmon [43] and eel, *Anguilla anguilla*, [44]. However, contrary to expectations, another study on lab-reared stickleback observed a higher expression of CFTR in freshwater compared to saltwater [19]. Further, when comparing long-term expression levels of CFTR in saltwater exposed Atlantic salmon, the expression levels tended to decline towards the control after 30 days [43]. Although we would have expected a higher difference in the expression of CFTR, especially between the two control groups (SS and FF), there is much confounding evidence of CFTR-expression across taxa [45]; reported expression of CFTR in freshwater spans from not expressed at all in Mosambique tilapia, *Oreochromis mossambicus* [46], diffuse in Killifish [47] to no change in expression in striped bass, *Morone saxatilis* [48], indicating an overall complicated involvement of CFTR in freshwater osmoregulation [47]. An alternative to differential expression of the ion-transporter CFTR could be a redistribution and reuse of CFTR proteins, as Marshall et al. [47] illustrated movement of the protein from an apical location in SW to a more diffuse and basolateral location in FW. This could also be the case for the stickleback in this study, as a rearrangement of CFTR-proteins and hence its activity state would not be picked up by the qPCR analysis.

### Much variation, but higher overall expression of KCNH4 in freshwater fish

Comparing the KCNH4-expression between the two control treatments (SS and FF), there was more temporal variation than would have been expected: KCNH4-expression decreased during the first 24 h in the SS treatment, whereas in the FF treatment the expression increased the first 48 h, before both seemed to be stabilized. The transfer of marine fish to freshwater (SF) followed approximately the same curve as for the SS treatment, and the transfer of freshwater fish to saltwater (FS) also demonstrate a down-regulated expression during the first 24 h, before expression increased reaching control levels (5 min exposure) after three weeks. Overall, the expression was higher in FF and FS compared to SS and SF. However, as there was no overall trend in the expression of KCNH4 for any of the groups in this study, this indicates that the gene is involved in other processes than osmoregulation, or that the osmoregulatory function of this transcript is located in other organs than the gill.

KCNH4 is a voltage-gated ion channel protein that is sensitive to voltage changes in the cell membrane and is known to have several functions, including regulation of cell volume, maintaining resting currents and affecting cardiac contractility [49]. Recent genome re-sequencing of 21 individual stickleback from marine and freshwater habitats across their global distribution revealed 81 loci underlying repeated parallel divergence in marine and freshwater ecotypes, including three chromosomal inversions [20]. One of the inversion sites has marine and freshwater specific 3′ exons of the KCNH4-gene, indicating parallel ecological selection on the breaking sites of the inversion, and possibly also directly on KCNH4 [20]. Although it is clear that chromosomal rearrangements can contribute to speciation [50], [51], [52], it is less evident how they do so and which mechanisms are involved. It is therefore important to identify whether inversion events have led to profound changes in the expression pattern of genes involved in the inversion, in relevant experimental setups.

While we do not have any information on the chromosomal arrangements in these two populations, it is still interesting to quantify the expression of the gene across different environments, as one would expect different expression profiles if the gene is under osmoregulatory selection. Chromosomal inversions are known to alter gene activity, either by causing non-functionalization of the gene, generating alternative splice sites or by altering gene regulatory networks [53], [54]. In a study on development under different thermal selection regimes, one population of *Drosophila subobscura* had different expression patterns for loci located within and between inversions, where significant differences in expression tended to be more commonly found inside rather than outside the inversion [55]. The increased expression of KCNH4 in freshwater found in this study indicates a potential regulatory effect of the inversion on chromosome 1, however more studies are needed in order to disentangle the complete effect of the inversion.

### Expression of HSP70 was elevated in freshwater adapted fish

HSP70 was identified as a candidate gene for detecting short-term osmotic stressful conditions in stickleback, with higher expression in freshwater compared to saltwater. When comparing SS and FF expression of HSP70, the marine fish had an overall lower expression before stabilizing after the first 24 h. Marine fish exposed to freshwater (SF) had an increased expression compared to SS the first 6 h before normalizing and freshwater fish exposed to saltwater (FS) had an overall lower expression compared to FF.

Capture, handling and crowding are all factors that can initiate a stress response in fish, as can short-term fluctuations in the physical environment. Physiological responses to stressors are complex, but include increased activity of cellular defense mechanisms, such as the up-regulation of HSP-genes [56]. The involvement of HSP70 in the acclimation of fish to salinity changes has been well documented experimentally [57], [58], [59]. Larsen et al. [59] observed a significant induction of HSP70 in kidney tissue of two populations of flounder, *Platichtys flesus*, when introduced to non-native salinities for both short- and long term exposures. However, the same study also illustrated tissue-specific up-regulation of HSP70, as expression in gill and liver was differentially affected by differences in salinity [59]. Other similar studies on Atlantic cod, *Gadus morhua*, illustrated expression differentiations in both gills and kidney after salinity transplantations during the first 24 hours [60], similar to the result in this study.

### Conclusions and perspectives

In our study system, sticklebacks from the marine population are genetically (Østbye et al., unpublished data) and morphologically [61] differentiated from the freshwater population living in the river below the waterfall. However, as the populations are genetically differentiated, a surprising result of this study is how little variation in gene expression was observed when the fish were directly transferred to the contrasting salinity treatments, and additionally, how little it differed between the two populations in their native salinities.

Movement between environments of different salinity is physiologically costly [62], and resident populations experiencing different salinity levels are predicted to be locally adapted to their native habitat as traits promoting euryhalinity are expected to be rapidly lost if they are not under selection [63]. It is likely that adaption to the local environment takes time, but the freshwater fish in this study have been separated from the marine populations for more than 7000 years (between 3500 to 7000 generations assuming a two year or one year life cycle), likely sufficient time for local adaptation given reasonable selection [64], [65]. Further, population genetic studies on stickleback have recognized salinity to be a major factor in the distribution of genotypes in systems that exchange migrants [66], [67], also across high gene-flow environments such as in the Baltic ocean [68], indicating that adaption to salinity is under selection.

Based on survival alone, the results from this study suggest that over the>3500 generations of adaptions to freshwater environments, the osmoregulatory physiology of landlocked stickleback has not been significantly canalized or experienced strong selection as they have retained their capacity for osmoregulation in saltwater. Additionally the locally adapted marine stickleback can osmoregulate in freshwater, despite originating as a marine species. This indicates that for stickleback, the cost of retaining osmoregulatory plasticity is small, or that the traits promoting euryhalinity in stickleback are under strong selection or pleitropically linked to other traits under strong selection. However, for fish living in marine and freshwater environments, the selection pressure for osmoregulation is still opposite, indicating that the stickleback must have alternative cell-regulating mechanisms for survival in unfamiliar salinities. In this study we only targeted gene expression values by quantifying the amount of mRNA extracted from gill tissue, however, the amount of mRNA does not necessarily imply equal concentrations of the functional proteins as a number of mechanisms can limit or increase the production. Protein expression profiles, also from kidney tissue, could have revealed different results. Other alternative strategies the stickleback may be utilizing could include changes in activity state of the ion-transport proteins by movement within the cell (activation/down-regulation), or by re-using the proteins by reversing the orientation in the cell-membrane, or by modulations following the interaction of other proteins [69], [70], [71].

Whatever method it is that the stickleback seems to be employing to osmoregulate so effectively, it has created a species that is incredibly well able to colonize new habitats regardless of the salinity they find themselves in, which has been a huge asset to this species in its spread throughout the northern hemisphere. Additional studies targeting the exact genetic and physiological mechanisms for the wide salinity tolerance in marine and freshwater stickleback are needed to understand the stickleback's incredible capacity for ion secretion and absorption. Their ability to adapt immediately to the environmental demands, with no apparent increase in physiological stress, is as unusual as it is intriguing, especially as this has been so evolutionarily important for this widespread species.

## Supporting Information

Appendix S1 **The Cq-values for the four targeted genes after normalization, ATP1A3, CFTR, KCNH4 and HSP70 in saltwater control (SS, dark orange), saltwater fish exposed to freshwater (SF, light orange), freshwater control (FF, dark blue) and freshwater fish exposed to saltwater (FS, light blue) for the nine different exposure periods.** Higher values indicate lower expression values.
(EPS)

## References

[1] West-Eberhard MJ (2005) Phenotypic accommodation: adaptive innovation due to developmental plasticity. Journal of Experimental Zoology Part B-Molecular and Developmental Evolution 304B: 610–618. 10.1002/jez.b.21071 16161068

[2] DeWitt TJ, Sih A, Wilson DS (1998) Costs and limits of phenotypic plasticity. Trends in Ecology & Evolution 13: 77–81. 21238209 10.1016/s0169-5347(97)01274-3

[3] Masel J, King OD, Maughan H (2007) The loss of adaptive plasticity during long periods of environmental stasis. American Naturalist 169: 38–46. 10.1086/510212 PMC1766558 17206583

[4] Lande R (2009) Adaptation to an extraordinary environment by evolution of phenotypic plasticity and genetic assimilation. Journal of Evolutionary Biology 22: 1435–1446. 19467134 10.1111/j.1420-9101.2009.01754.x

[5] Schultz ET, McCormick SD (2013) Euryhalinity in an evolutionary context. In: McCormick SD, Farrell AP, Brauner CJ, editors. Fish physiology: Euryhaline fishes. Oxford: Elsevier Science. pp. 477–529.

[6] Edwards SL, Marshall WS (2012) Principles and patterns of osmoregulation and euryhalinity in fishes. In: Stephen D. McCormick APF, Colin JB, editors. Fish Physiology: Academic Press. pp. 1–44.

[7] Evans DH, Piermarini PM, Choe KP (2005) The multifunctional fish gill: dominant site of gas exchange, osmoregulation, acid-base regulation, and excretion of nitrogenous waste. Physiological Reviews 85: 97–177. 15618479 10.1152/physrev.00050.2003

[8] Varsamos S, Nebel C, Charmantier G (2005) Ontogeny of osmoregulation in postembryonic fish: A review. Comparative Biochemistry and Physiology a-Molecular & Integrative Physiology 141: 401–429. 10.1016/j.cbpb.2005.01.013 16140237

[9] Griffith RW (1974) Environment and salinity tolerance in the genus *Fundulus* . Copeia 1974: 319–331.

[10] Heuts MJ (1947) Experimental studies on adaptive evolution in *Gaserosteus-aculeatus L* . Evolution 1: 89–102.

[11] Wootton RJ (1976) The biology of the sticklebacks. New York: Academic Press.

[12] Bell MA (1977) Late Miocene marine Threespine stickleback, *Gasterosteus aculeatus*, and its zoogeographic and evolutionary significance. Copeia: 277–282.

[13] Bell MA, Foster SA (1994) The evolutionary biology of the threespine stickleback. New York: Oxford University Press.

[14] Grøtan K, Østbye K, Taugbøl A, Vøllestad LA (2012) No short-term effect of salinity on oxygen consumption in threespine stickleback (*Gasterosteus aculeatus*) from fresh, brackish, and salt water. Canadian Journal of Zoology 90: 1386–1393.

[15] Raeymaekers JAM, Maes GE, Audenaert E, Volckaert FAM (2005) Detecting Holocene divergence in the anadromous-freshwater three-spined stickleback (*Gasterosteus aculeatus*) system. Molecular Ecology 14: 1001–1014. 15773932 10.1111/j.1365-294X.2005.02456.x

[16] Von Hippel FA, Weigner H (2004) Sympatric anadromous-resident pairs of threespine stickleback species in young lakes and streams at Bering Glacier, Alaska. Behaviour 141: 1441–1464.

[17] McPhail JD (1994) Speciation and the evolution of reproductive isolation in the sticklebacks (*Gasterosteus*) of south-western British Columbia. In: Bell AM, Foster JR, editors. The evolutionary biology of the threespine stickleback. Oxford: Oxford University Press. pp. 399–437.

[18] Møller JJ (2003) Relative sea level change in Fennoscandia. Net version 3.00. University of Tromsø: Department of Geology, TMU.

[19] McCairns RJS, Bernatchez L (2010) Adaptive divergence between freshwater and marine sticklebacks: Insights into the role of phenotypic plasticity from an intergrated analysis of candidate gene expression. Evolution 64: 1029–1047. 19895556 10.1111/j.1558-5646.2009.00886.x

[20] Jones FC, Grabherr MG, Chan YF, Russell P, Mauceli E, et al (2012) The genomic basis of adaptive evolution in threespine sticklebacks. Nature 484: 55–61. 22481358 10.1038/nature10944 PMC3322419

[21] Whitehead A, Roach JL, Zhang SJ, Galvez F (2012) Salinity- and population-dependent genome regulatory response during osmotic acclimation in the killifish (*Fundulus heteroclitus*) gill. Journal of Experimental Biology 215: 1293–1305. 22442367 10.1242/jeb.062075

[22] Mobasheri A, Avila J, Cozar-Castellano I, Brownleader MD, Trevan M, et al (2000) Na+, K+-ATPase isozyme diversity; Comparative biochemistry and physiological implications of novel functional interactions. Bioscience Reports 20: 51–91. 10965965 10.1023/a:1005580332144

[23] Bonting SL (1970) Sodium-pottassium activated adenosine triphosphatase and cation transport. In: Bittar EE, editor. Membranes and ion transport. New York: John Wiley & Sons. pp. 257–363.

[24] Silva P, Solomon R, Spokes K, Epstein FH (1977) Ouabain inhibition of gill Na-K ATPase: relationship to active chloride transport. Journal of Experimental Zoology 199: 419–426. 139454 10.1002/jez.1401990316

[25] McCormick SD, Sundell K, Bjornsson BT, Brown CL, Hiroi J (2003) Influence of salinity on the localization of Na+/K+-ATPase, Na+/K+/2Cl(−)cotransporter (NKCC) and CFTR anion channel in chloride cells of the Hawaiian goby (*Stenogobius hawaiiensis*). Journal of Experimental Biology 206: 4575–4583. 14610041 10.1242/jeb.00711

[26] Scott GR, Richards JG, Forbush B, Isenring P, Schulte PM (2004) Changes in gene expression in gills of the euryhaline killifish *Fundulus heteroclitus* after abrupt salinity transfer. American Journal of Physiology-Cell Physiology 287: C300–C309. 15044150 10.1152/ajpcell.00054.2004

[27] Hoekstra HE (2006) Genetics, development and evolution of adaptive pigmentation in vertebrates. Heredity 97: 222–234. 16823403 10.1038/sj.hdy.6800861

[28] Chan YF, Marks ME, Jones FC, Villarreal G, Shapiro MD, et al (2010) Adaptive evolution of pelvic reduction in Sticklebacks by recurrent deletion of a Pitx1 enhancer. Science 327: 302–305. 20007865 10.1126/science.1182213 PMC3109066

[29] Rosenblum EB, Roempler H, Schoeneberg T, Hoekstra HE (2010) Molecular and functional basis of phenotypic convergence in white lizards at White Sands. Proceedings of the National Academy of Sciences of the United States of America 107: 2113–2117. 20080544 10.1073/pnas.0911042107 PMC2836677

[30] Sørensen J, Kristensen T, Loeschcke V (2003) The evolutionary and ecological role of heat shock proteins. Ecology Letters 6: 1025–1037.

[31] Sanogo YO, Hankison S, Band M, Obregon A, Bell AM (2011) Brain transcriptomic response of threespine sticklebacks to cues of a predator. Brain Behavior and Evolution 77: 270–285. 10.1159/000328221 PMC3182040 21677424

[32] Knag AC, Taugbøl A (2013) Acute exposure to offshore produced water has an effect on stress- and secondary stress responses in three-spined stickleback *Gasterosteus aculeatus* . Comparative Biochemistry and Physiology C-Toxicology & Pharmacology 158: 173–180. 10.1016/j.cbpc.2013.07.004 23916882

[33] Krogh A (1937) Osmotic regulation in fresh water fishes by active absorption of chloride ions. Zeitschrift vergleichende Physiologie 24: 656–666.

[34] Evans DH (2008) Teleost fish osmoregulation: what have we learned since August Krogh, Homer Smith, and Ancel Keys. American Journal of Physiology-Regulatory Integrative and Comparative Physiology 295: R704–R713. 10.1152/ajpregu.90337.2008 18525009

[35] Livak KJ, Schmittgen TD (2001) Analysis of relative gene expression data using real-time quantitative PCR and the 2(T)(-Delta Delta C) method. Methods 25: 402–408. 11846609 10.1006/meth.2001.1262

[36] Bystriansky JS, Schulte PM (2011) Changes in gill H+-ATPase and Na+/K+-ATPase expression and activity during freshwater acclimation of Atlantic salmon (*Salmo salar*). Journal of Experimental Biology 214: 2435–2442. 21697436 10.1242/jeb.050633

[37] Shrimpton JM, Patterson DA, Richards JG, Cooke SJ, Schulte PM, et al (2005) Ionoregulatory changes in different populations of maturing sockeye salmon *Oncorhynchus nerka* during ocean and river migration. Journal of Experimental Biology 208: 4069–4078. 16244166 10.1242/jeb.01871

[38] Jensen MK, Madsen SS, Kristiansen K (1998) Osmoregulation and salinity effects on the expression and activity of Na+, K+-ATPase in the gills of European sea bass, *Dicentrarchus labrax* (L.). Journal of Experimental Zoology 282: 290–300. 9755480 10.1002/(sici)1097-010x(19981015)282:3<290::aid-jez2>3.0.co;2-h

[39] Stagg RM, Shuttleworth TJ (1982) Na+, K+ ATPase, quabain binding and quabain-sensitive oxygen consumption in gills from*Platichthys flesus* adapted to seawater and freshwater. Journal of comparative physiology 147: 93–99.

[40] Folmar LC, Dickhoff WW (1980) The parr-smolt transformation (smoltification) and seawater adaptation in salmonids: A review of selected literature. Aquaculture 21: 1–37.

[41] Perry SF (1997) The chloride cell: Structure and function in the gills of freshwater fishes. Annual Review of Physiology 59: 325–347. 10.1146/annurev.physiol.59.1.325 9074767

[42] Kirschner LB (2004) The mechanism of sodium chloride uptake in hyperregulating aquatic animals. Journal of Experimental Biology 207: 1439–1452. 15037638 10.1242/jeb.00907

[43] Singer TD, Clements KM, Semple JW, Schulte PM, Bystriansky JS, et al (2002) Seawater tolerance and gene expression in two strains of Atlantic salmon smolts. Canadian Journal of Fisheries and Aquatic Sciences 59: 125–135.

[44] Wilson JM, Antunes JC, Bouca PD, Coimbra J (2004) Osmoregulatory plasticity of the glass eel of *Anguilla anguilla*: freshwater entry and changes in branchial ion-transport protein expression. Canadian Journal of Fisheries and Aquatic Sciences 61: 432–442.

[45] Havird JC, Henry RP, Wilson AE (2013) Altered expression of Na+/K+-ATPase and other osmoregulatory genes in the gills of euryhaline animals in response to salinity transfer: A meta-analysis of 59 quantitative PCR studies over 10 years. Comparative Biochemistry and Physiology D-Genomics & Proteomics 8: 131–140. 10.1016/j.cbd.2013.01.003 23466469

[46] Hiroi J, McCormick SD, Ohtani-Kaneko R, Kaneko T (2005) Functional classification of mitochondrion-rich cells in euryhaline Mozambique tilapia (*Oreochromis mossambicus*) embryos, by means of triple immunofluorescence staining for Na/K+-ATPase, Na+/K+/2Cl(−) cotransporter and CFTR anion channel. Journal of Experimental Biology 208: 2023–2036. 15914646 10.1242/jeb.01611

[47] Marshall WS, Lynch EA, Cozzi RRF (2002) Redistribution of immunofluorescence of CFTR anion channel and NKCC cotransporter in chloride cells during adaptation of the killifish *Fundulus heteroclitus* to sea water. Journal of Experimental Biology 205: 1265–1273. 11948203 10.1242/jeb.205.9.1265

[48] Madsen SS, Jensen LN, Tipsmark CK, Kiilerich P, Borski RJ (2007) Differential regulation of cystic fibrosis transmembrane conductance regulator and Na+, K+-ATPase in gills of striped bass, *Morone saxatilis*: effect of salinity and hormones. Journal of Endocrinology 192: 249–260. 17210762 10.1677/JOE-06-0016

[49] Gutman GA, Chandy KG, Grissmer S, Lazdunski M, McKinnon D, et al (2005) International union of pharmacology. LIII. Nomenclature and molecular relationships of voltage-gated potassium channels. Pharmacological Reviews 57: 473–508. 16382104 10.1124/pr.57.4.10

[50] Ellegren H, Smeds L, Burri R, Olason PI, Backstrom N, et al (2012) The genomic landscape of species divergence in *Ficedula* flycatchers. Nature 491: 756–760. 23103876 10.1038/nature11584

[51] Rieseberg LH (2001) Chromosomal rearrangements and speciation. Trends in Ecology & Evolution 16: 351–358. 11403867 10.1016/s0169-5347(01)02187-5

[52] Noor MAF, Grams KL, Bertucci LA, Reiland J (2001) Chromosomal inversions and the reproductive isolation of species. Proceedings of the National Academy of Sciences of the United States of America 98: 12084–12088. 11593019 10.1073/pnas.221274498 PMC59771

[53] Kirkpatrick M, Barton N (2006) Chromosome inversions, local adaptation and speciation. Genetics 173: 419–434. 16204214 10.1534/genetics.105.047985 PMC1461441

[54] Matzkin LM, Merritt TJS, Zhu CT, Eanes WF (2005) The structure and population genetics of the breakpoints associated with the cosmopolitan chromosomal inversion In(3R)Payne in *Drosophila melanogaster* . Genetics 170: 1143–1152. 15781702 10.1534/genetics.104.038810 PMC1451188

[55] Laayouni H, Garcia-Franco F, Chavez-Sandoval BE, Trotta V, Beltran S, et al. (2007) Thermal evolution of gene expression profiles in Drosophila subobscura. Bmc Evolutionary Biology 7.. 10.1186/1471-2148-7-42 PMC1847442 17371595

[56] Moseley P (2000) Stress proteins and the immune response. Immunopharmacology 48: 299–302. 10960671 10.1016/s0162-3109(00)00227-7

[57] Deane E, Kelly S, Luk J, Woo N (2002) Chronic salinity adaptation modulates hepatic heat shock protein and insulin-like growth factor I expression in black sea bream. Marine Biotechnology 4: 193–205. 14961280 10.1007/pl00021690

[58] Fangue NA, Hofmeister M, Schulte PM (2006) Intraspecific variation in thermal tolerance and heat shock protein gene expression in common killifish, Fundulus heteroclitus. Journal of Experimental Biology 209: 2859–2872. 16857869 10.1242/jeb.02260

[59] Larsen PF, Nielsen EE, Williams TD, Loeschcke V (2008) Intraspecific variation in expression of candidate genes for osmoregulation, heme biosynthesis and stress resistance suggests local adaptation in European flounder (*Platichthys flesus*). Heredity 101: 247–259. 18560442 10.1038/hdy.2008.54

[60] Larsen PF, Nielsen EE, Meier K, Olsvik PA, Hansen MM, et al (2012) Differences in salinity tolerance and gene expression between two populations of Atlantic Cod (*Gadus morhua*) in response to salinity stress. Biochemical Genetics 50: 454–466. 22205502 10.1007/s10528-011-9490-0

[61] Bjærke O, Østbye K, Lampe HM, Vøllestad LA (2010) Covariation in shape and foraging behaviour in lateral plate morphs in the three-spined stickleback. Ecology of Freshwater Fish 19: 249–256.

[62] Moyle PB, Cech JJ (1996) Fishes. An introduction to ichthyology. 3rd edition. Upper Saddle River, N.J.: Prentice Hall.

[63] Schultz ET, McCormick SD (2012) Euryhalinity in an evolutionary context. In: Stephen D. McCormick APF, Colin JB, editors. Fish physiology: Academic Press. pp. 477–533.

[64] Kinnison MT, Hendry AP (2001) The pace of modern life II: from rates of contemporary microevolution to pattern and process. Genetica 112: 145–164. 11838763

[65] Hendry AP, Kinnison MT (1999) Perspective: The pace of modern life: Measuring rates of contemporary microevolution. Evolution 53: 1637–1653. 28565449 10.1111/j.1558-5646.1999.tb04550.x

[66] McCairns RJS, Bernatchez L (2008) Landscape genetic analyses reveal cryptic population structure and putative selection gradients in a large-scale estuarine environment. Molecular Ecology 17: 3901–3916. 18662229 10.1111/j.1365-294X.2008.03884.x

[67] Taugbøl A, Junge C, Quinn TP, Herland A, Vøllestad LA (2014) Genetic and morphometric divergence in threespine stickleback in the Chignik catchment, Alaska. Ecology and Evolution 4: 144–156. 24558570 10.1002/ece3.918 PMC3925378

[68] DeFaveri J, Jonsson PR, Merilä J (2013) Heterogeneous genomic differentiation in marine threespine stickleback: Adaptation along an environmental gradient. Evolution 67: 2530–2546. 24033165 10.1111/evo.12097

[69] Pertl H, Pockl M, Blaschke C, Obermeyer G (2010) Osmoregulation in Lilium pollen grains occurs via modulation of the plasma membrane H+ ATPase activity by 14-3-3 proteins. Plant Physiology 154: 1921–1928. 20974894 10.1104/pp.110.165696 PMC2996032

[70] Szczesnaskorupa E, Browne N, Mead D, Kemper B (1988) Positive charges at the NH2 terminus convert the membrane-anchor signal peptide of cytochrome P-450 to a secretory signal peptide. Proceedings of the National Academy of Sciences of the United States of America 85: 738–742. 3422456 10.1073/pnas.85.3.738 PMC279630

[71] Hartmann E, Rapoport TA, Lodish HF (1989) Predicting the orientation of eukaryotic membrane-spanning proteins. Proceedings of the National Academy of Sciences of the United States of America 86: 5786–5790. 2762295 10.1073/pnas.86.15.5786 PMC297715

